# Experimental design of Al_2_O_3_/MWCNT/HDPE hybrid nanocomposites for hip joint replacement

**DOI:** 10.1080/21655979.2020.1775943

**Published:** 2020-06-16

**Authors:** Sameh Dabees, Bahaa M. Kamel, Vineet Tirth, Abou Bakr Elshalakny

**Affiliations:** a Department of Engineering, Klaipeda University, Klaipeda, Lithuania; b Mechanical Engineering Department, National Research Centre, Giza, Egypt; c Mechanical Engineering Department, College of Engineering, King Khalid University, Asir, Kingdom of Saudi Arabia; d Research Center for Advanced Materials Science (RCAMS), King Khalid University, Asir, Kingdom of Saudi Arabia; e Production Engineering and Printing Technology Department, Akhbar El Yom Academy, Giza, Egypt

**Keywords:** Hip joint replacement, HDPE, MWCNTs, nanocomposites, Al_2_o_3_, TGA; biomaterials

## Abstract

Fracture in the hip joint is a major and quite common health issue, particularly for the elderly. The loads exploited by the lower limbs are very acute and severe; in the femur, they can be several folds higher than the whole weight of the body. Nanotechnology and nanocomposites offer great potential in biomedical applications. The organic materials are more biocompatible. Mechanical properties like strength and hardness are challenging parameters which control the selection of a joint. HDPE in its pure form has been successfully used as a prosthetic foot (external) but failed as an implant material due to limited mechanical properties. High-density polyethylene thermoplastic polymer (HDPE) and multi-walled carbon nanotubes (MWCNT)/Nano-Alumina is selected as a potential material for a biomedical implant and its mechanical properties and biocompatibility have been discussed. HDPE/MWCNT/Alumina (Al_2_O_3_) nanocomposites have not been explored yet for prosthetic implants. These nanocomposites were prepared in this investigation in different compositions. Prepared material has been physiochemically characterized to check the morphology and the structure. MWCNTs enhanced hardness and elastic modulus of the HDPE. Optimization of the material composition revealed that hybrid composite with structure (2.4% Al_2_O_3_ and 0.6% MWCNT) exhibits better mechanical properties compared to other ratios with 3% MWCNTs and 5% MWCNTs. Thermal gravimetric analysis (TGA) dedicates that the percentage of crystallization has been increased to 6% after adding MWCNT to HDPE. The moisture absorption decreased to 90% with 5% MWCNT. Experimental results of Colorimetric assay (MTT) of a normal human epithelial cell line (1- BJ1) over Al_2_O_3_/MWCNT@HDPE showed <20% cytotoxic activity, proving its acceptance for medical use. HDPE/MWCNT/Al_2_O_3_ nanocomposites emerged as a candidate material for artificial joints.

## Graphical abstract

**Figure:**
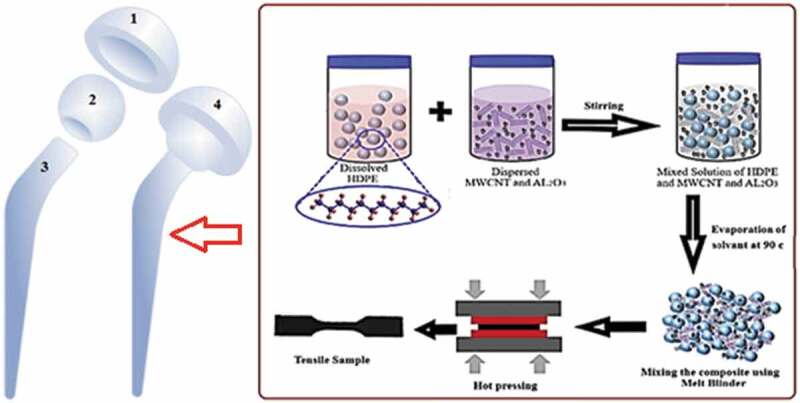

## 1. Introduction

Hip joint represents pose a major health challenge to medical fraternity nowadays. The hip prosthesis is a key factor in the selection of biomaterials and the fabrication of replacement joints to meet the requirements of arthroplasty. Massive loads experienced by the limbs pose another challenge. Abnormal combinations of mechanical and physical characteristics are the main parameters that control the quality and feasibility of the hip joint. While joint replacement represents attractive cases in the specialization of orthopedic surgery but maintaining the design and structure for a long time is still a big challenge. The mean half-life time of the hip joint is usually among the range of 15–20 years. High strength, toughness, tailored stiffness, resistance to impact and corrosion, and biocompatibility are critical for tissue substitute structures. Widely used prosthetic materials include ceramics and metals. The metals and alloys are typically used for fracture support, facial and dental restoration, and stents. The biomedical-grade Ti alloys are widely preferred as a load-bearing implant material. The Ti-alloys exhibit excellent tribological properties but they have certain drawbacks such as high cost and weight. There are several other low-cost metals and alloys, which are also used as implant materials [1]. These metals release some toxins over a period questioning their biocompatibility [2]. The ions and the debris generated from metallic materials during bio-tribocorrosion are hazardous for the health of the patient. The mechanical wear and high chemical activity resulting in corrosion, are responsible for producing hazardous debris [3]. The ceramic materials are typically used in orthopedic and dental reconstruction as well as making synthetic valves. Among inorganic implant materials for hip joint replacement, a combination of alumina and silicon nitride exhibited less wear for physically challenging gait requirements [4]. Poor ductility and high brittleness is a limiting factor in the application of ceramic materials in the prosthetic application.

Organic materials, largely polymers are now preferred for such applications due to a wide classification of chemical compositions, characteristics, feasibility, and biocompatibility [5–7]. While polymers are typically used for grafts, catheters, tendons, and softer tissue restoration. In recent times, the less weight and biocompatibility of polymers have led to their use in prosthetic applications but their low strength has been a limiting factor. Among polymers, ultra-high molecular weight polyethylene (UHMWPE) and its composites were successfully used as a prosthetic hip joint material. UHMWPE exhibits acceptable cytocompatibility, mechanical and tribological properties [2]. Hydroxyapatite (Ca_5_(OH)(PO_4_)_3_) known as HA, constitutes 70% of human bones. It is a primary mineral present in bones and teeth. The HA nanorods and nanoplates were recommended for the biomedical applications [8]. HDPE exhibits good mechanical behavior includes high strength to density ratio, lightweight, and chemical resistance. However, it possesses low hardness and is unfit for prolonged use [9–13]. A few studies attempted to improve the properties of HDPE by adding various types of fillers such are talc, nano-hydroxyapatite, and nano aluminum oxide [14,15]. The results displayed an initial increase in the degree of crystallinity and modulus of the composites, followed by a decrease at higher ratios [16,17]. Also, the friction coefficient of composites reduced, and the hardness increased with the increment of carbon fibers [18,19]. HDPE/Hydroxyapatite (HA) composites have been assembled and commercialized under the name of HAPEX™, showing very promising mechanical properties alike human bone [20,21]. It was observed that by replacing the HA content with alumina, the tribological characteristics of HAPEX™ improved significantly [22,23]. The HDPE was used as a total hip joint replacement material. The clinical observations showed that the joint loosened over long-term use and resulted in the ultimate failure of the arthroplasty. Thus, HDPE in its pure form was not recommended for hip joint implants requiring challenging gait necessities [24]. However, HDPE has been used for the prosthetic foot, fixed externally with performance conformance of 94%, and the patient satisfaction of 85% [25] .

Since the great discovery of polymer composites by the Toyota research group [26], a new track of research has flourished in the field of joint replacement. Previously, researchers used to apply inorganic nanomaterials as load-bearing components in the preparation of composites with very high processing and manufacturing costs [27]. Furthermore, preparing composites based on inorganic compounds may produce toxic materials, which are not suitable for medical use. Nanotechnology provides a new opportunity to improve the performance of the composites together with the carbon compounds because of the unique characteristics of the nanoparticles. Nanocarbon materials like fullerene, carbon nanofibers and carbon nanotubes have earned great attention in polymer science. Carbon nanotubes or single layers of graphene rolled into a cylinder shape, have been commonly applied and evolved into diverse research directions including medical applications. CNT usually exists in 2 phases, single-walled and multiwalled, with different characteristics properties [28–33]. There are different types of techniques that have been improved for synthesizing CNT which mainly involve vapor phase routes. Commonly, CNTs are produced by chemical vapor deposition, laser ablation, and carbon arc-discharge processes [34]. The amalgamation of carbon nanostructures and polymer matrices were widely studied to adjust the mechanical properties and to extend their applications [35,36]. Recently, carbon nanotubes and graphite were excessively used to develop composites for different applications [37–48]. MWCNT have truly superior mechanical, electrical properties. For example, they are stronger then steal, much better conductors then copper. Their Young’s modulus is over 1 TPa and their tensile strength is estimated to be 10–60 GPa so by adding MWCNTs will affect the mechanical properties and improve the hardness as reported in many articles [49–52].

The exploration of HDPE as a potential substrate for nanocoating is a possibility that might be studied. But the basic requirement of a sustainable load-bearing joint includes its properties from the core and its biocompatibility. The science and technology is ever aspiring to explore superior materials with lesser liabilities.

The HDPE is light in weight than most metallic and ceramic implants but it is not suitable in its pure form for a load-bearing prosthetic implant. Alumina has been successfully used as a constituent of the ceramic implant. The present prosthetic implant materials offer a solution but come with side effects and limitations. After the successful application of UHMWPE for prosthetic implants and HDPE for external prosthetic limbs and the fact that the metallic implants have their own hazardous and the ceramic implants have limitations, it is worth investigating to explore the possibility of HDPE based nanocomposites for the prosthetic implants. The research design of the present study includes the preparation of an HDPE based nanocomposite, which includes the preparation of the nanocomposite with a limited amount of MWCNT and alumina and their characterization. The cytotoxicity of the selected nanocomposites has been studied to determine their biocompatibility. Such nanocomposites are expected to provide a low cost and lightweight nontoxic alternative to the conventional hip joint materials for challenging gait requirements. This research focuses mainly on studying the effect of impeding MWCNT inside the pores as well as over the surface of HDPE polymer. In this research work, different ratios of MWCNT were incorporated inside the matrix of HDPE to improve the hardness and other mechanical properties of polymer from the perspective of hip joint application. Al_2_O_3_/MWCNT@HDPE nanocomposite was prepared by the wet chemical method. The nanocomposite was characterized to examine the structure and morphology. MWCNT act as solid lubricants and are used in wear-resistant applications such as bearing materials. The same properties of MWCNT were exploited in this study to improve the overall mechanical properties of HDPE. Also, the influence of adding MWCNT over the normal cell (Cytotoxic activity test) was studied to determine its feasibility for medical applications.

## 2. Materials and methods

High-density polyethylene (tensile strength-27.5 MPa and density-0.944 g/cm^3^) was purchased from Nanotech company. MWCNTs and nano alumina (Al₂O₃) used in this research were purchased from Middle east company for the petrochemical industry. The MWCNT had 8–13 nm diameter, 3–15 μm length, and 99.87% purity. The physical properties of nano-Al₂O₃ are density at RT 3.9 g/cm^3^, particle size 20–30 nm, and 99.95% purity.

**Figure 1.**
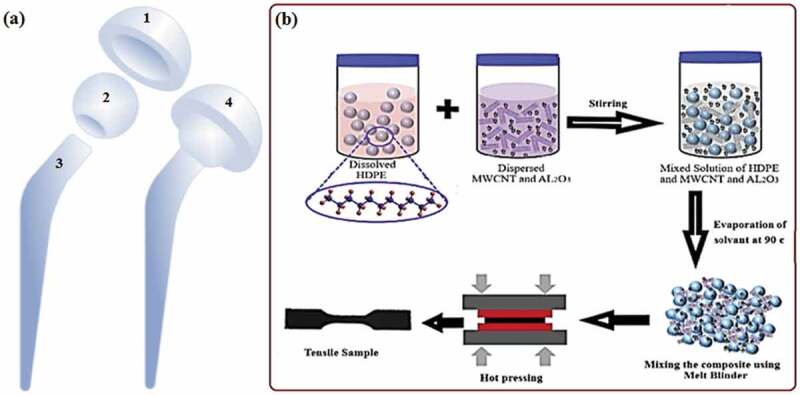
(a) Formation of a hip joint, 1-acetabular cup, 2-femoral head ball, 3-femoral stem, 4-assembled hip joint. (b) Schematic diagram of HDPE/MWCNT/Al_2_O_3_ manufacturing process.

The formation of a prosthetic hip joint is presented in Figure 1(a). The major components of a prosthetic hip joint are acetabular cup, femoral head ball, and femoral stem. A liner is also used sometimes between the acetabular cup and the femoral head. The first composition of MWCNT@ HDPE and the second composition of Al_2_O_3_/MWCNT@ HDPE composite were prepared by wet chemical approach as presented in Figure 1(b).

In particular uniform and reproducible dispersion in liquid phases considered one of the most important challenges in developing polymer nanocomposite applications. The dispersion process was done in the presence of organic solvent ethanol (absolute C_2_H_2_OH). Polymer nanocomposite dispersed with ethanol was sonicating using digital Ultrasonic cleaning which utilizes cavitation bubbles incited by high recurrence (sound) waves to shake a fluid. The tumult creates the clinging of contaminants to the substrates. The sonicating time was fixed at 35 min. The composites were stirred using MS300HS hot stirrer. The time for stirring was fixed at 15 min [53,54]. The composites were dried using Dreier (Heraeus UT 6050) recirculated drying oven at a constant temperature to 250 °C for 45 min to evaporate the organic solvent (ethanol) [55,56].

Thermally conductive nano composites were manufactured by mixing MWCNT and HDPE using internal mixing (Melt blinding process). The mixing chamber contains two cylindrical screw rollers for melting and mixing. The internal mixer can rotate up to 2500 rpm powered by a motor of 7.5 hp. The polymer nano composite were melted in the internal mixer at a temperature of 185°C for about 20 min. The weight concentration of the MWCNT in the polymer matrix was initially 0, 1, 2, 3, and 5 %. Following the extraction of HDPE/MWCNT composite from the inward blender, hot pressing was done to transform the specimen into the desired shape. The dog bone type ASTMD-638-IV specimen were employed using two types of molds for the thermal conductivity and tensile strength tests, respectively. This process was done at a temperature of 190°C and a pressure of 150 bar. The total pressing time of specimen was about 45 min including time for holding the sample to cool them for 15 min. The specimens were cooled to the room temperature to enable crystallization and prevent the reappearance of the amorphous phase. HDPE/Al_2_O_3_/MWCNTS were produced by the same process as above.

Then the composite was stirred for 1 hour. Thereafter, the powder was separated and dried to remove the excess ethanol and the surfactant by maintaining a temperature of 185°C for about 120 min. Following the extraction of Al_2_O_3_/MWCNT@HDPE nanocomposite from the inward blender, hot pressing was done to obtain the desired shape of the specimens. Then, the specimens cooled rapidly to room temperature.

### 2.1. Material characterization

X-ray diffraction (XRD) technique was utilized to check the composition of phase material and the crystallinity of the prepared material [57]. The XRD measurement was performed using (Siemens XRD D5000) and a copper X-ray source within the range from 0° to 70°. The voltage and current were adjusted at 40 kV and 50 mA, respectively. The morphology and microstructure characterization of Al_2_O_3_/MWCNT@HDPE hybrid composites were studied using SEM and TEM systems [58–60].

Mechanical testing is applied to examine the characteristics like hardness, modulus, fracture toughness, and yield strength, etc. Uniaxial compression and tensile testing are typically measured to investigate the acquired elastic modulus [61]. The hardness test employs an indenter probe that is located over a surface under a specific weight load. Usually, the size of the indentation is calculated to determine hardness. Microhardness testing is considered as an acceptable industry standard for quality control for hardness data [62]. The Vickers microhardness tests were conducted to investigate the influence of MWCNTs. The total load is normally applied between 10 and 15 seconds. The zone of the slanting surfaces of the indentation was investigated and the Vickers hardness which is calculated by dividing the weight load (kg) with the area of indentation (mm^2^) [63]. Tensile measurements were performed on a universal testing machine with a speed of 5 mm/min. Percentages of the MWCNT were maintained between 1–5%. All samples were tested in the same environmental conditions.

Thermal gravimetric analysis (TGA) test estimates the weight change of a material, either as an element of expanding temperature, or isothermally as a function of time in the environment of nitrogen or helium. The degeneration behavior of polymers and their hybrid composites is usually determined by 3 parameters: the temperature at which the framework starts to debase, the degradation temperature T_d_, and the degradation rate Dr, which is found in the subsidiary weight reduction as an element of temperature curve [64,65]. TGA analysis was performed using (DTG −60 SIMULTANEOUSDTG- TG SHIIMAADZU, JAPAN) for each sample and HDPE/MWCNT/Al_2_O_3_ nanocomposite. The specimens were heated from 25°C to 750°C at a rate of 10°C/min in a nitrogen atmosphere. Flexural and mechanical characteristics usually decrease as the water absorption ratio increases. Tensile modulus was found to decrease at a higher level of water absorption, which is considered as a sensitive property. Therefore, it is important to investigate the effect of adding MWCNT over the moisture absorption of HDPE.

Water absorption examination (ASTM D750-95) has been carried out by a total inundation of 5 samples in distilled water at room temperature for 72 h. The water absorption was calculated by weighing the samples at time intervals. Since this application is intended for medical use, it is very important to check whether the material is feasible to be used inside the human body. Cell viability test was dedicated by the mitochondrial-dependent reduction of yellow MTT (3-(4,5-dimethylthiazol-2-yl)-2,5-diphenyl tetrazolium bromide) or MTT essay to magenta formazan [66].

All measurements were done in an antiseptic zone applying a laminar flux cabinet conforming to the biosafety class II level (Baker, SG403INT, Sanford, ME, USA). Cells were suspended in dulbecco’s modified eagle medium (DMEM-F12) with 1% antibiotic-antimycotic mixture (10,000 U/ml Potassium Penicillin, 10,000 µg/ml Streptomycin Sulfate and 25 µg/ml Amphotericin B) and 1% L-glutamine at 37°C under 5% CO_2_. Cells were refined for 5 days and then implanted at a concentration of 10*10^3^ cells/well in unprecedented plenary outgrowth medium, in 96-well microtiter plastic plates. After 48 h of incubation, the medium was aspirated, 40 ul MTT salt (2.5 μg/ml) was added to each well and hatched for further four hours at the same conditions [67,68]. Absorbance was estimated using a microplate multi-well reader (Bio-Rad Laboratories Inc., model 3350, Hercules, California, USA) at 595 nm at a reference wavelength of 620 nm.

## 3. Results and discussion

### 3.1. Characterization

**Figure 2.**
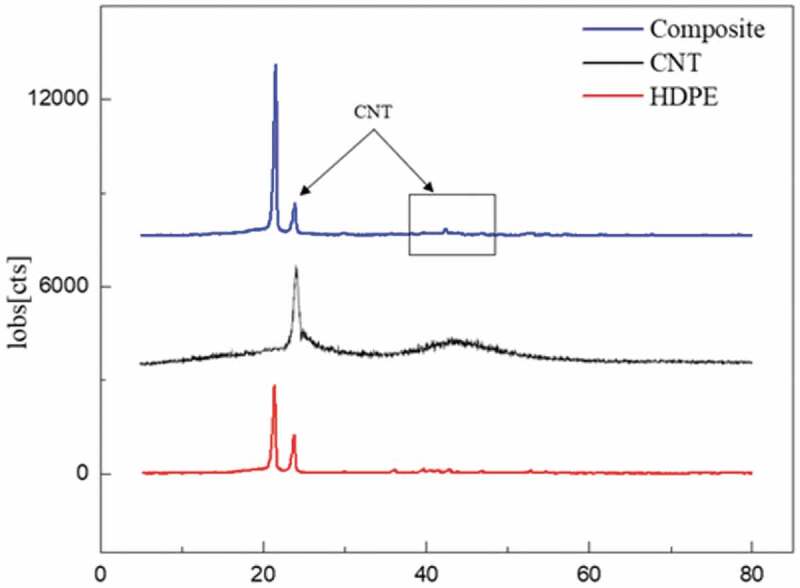
XRD patterns of HDPE, MWCNT, and HDPE/MWCNT hybrid composite.

The HDPE/MWCNT nanocomposites have been fabricated and characterized by X-ray diffraction, scanning electron microscope (SEM), and Transmission electron microscopy (TEM). Figure 2 dedicates the XRD diffraction pattern of HDPE, MWCNT, and HDPE/MWCNT hybrid nanocomposite. HDPE presented with two characteristic peaks at 21.4° and 23.2°. These peaks were also detected in the hybrid nanocomposite. The intensity of the peaks in the hybrid nanocomposite is greater ascribed to the increase in crystallinity. This could happen as a result of the interaction and crystallization behavior of MWCNT with HDPE during the blending process. The XRD pattern of MWCNTs is shown as well in Figure 2. MWCNT exhibits 2 peaks at around 2θ = 26° and other broad peak centered at 2θ = 43° corresponding to the (002) and (100) Bragg reflection planes having the interlayer spacing of 3.213 and 2.012 Å respectively [69]. The presence of MWCNT is acknowledged by the XRD diffraction pattern.

**Figure 3.**
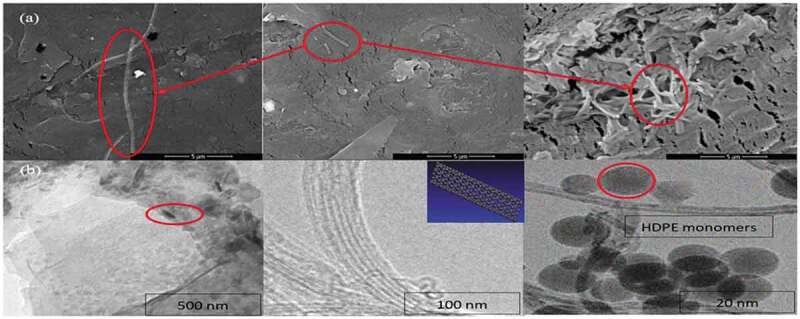
(a) SEM images of HDPE/MWCNT hybrid composite with 5% MWCNT ratio nanocomposite and (b) TEM of the same composite.

The images obtained from the transmission and scanning electron microscopes to explore the nanocomposite structure and the matrix distribution are shown in Figure 3. Scanning electron pictures Figure 3(a) of the nanocomposite samples clearly show the MWCNT among the polymer matrix. In the processing of HDPE and MWCNT blends by the melt blinder, the polymer chains of HDPE are overlapped with each other as observed in the SEM figures. The TEM images are shown in Figure 3(b) confirm the MWCNT structure and the nano-scaled design of the material. The uniform dispersion of MWCNT in a polymer matrix is a cumbersome task. The fairly homogenous distribution of the MWCNT over the whole matrix can be also observed indicating satisfactory suspension of the MWCNT in the HDPE. Hence the wet chemical synthesis of HDPE/MWCNT nanocomposites has been successfully achieved.

### 3.2. Mechanical properties

**Figure 4.**
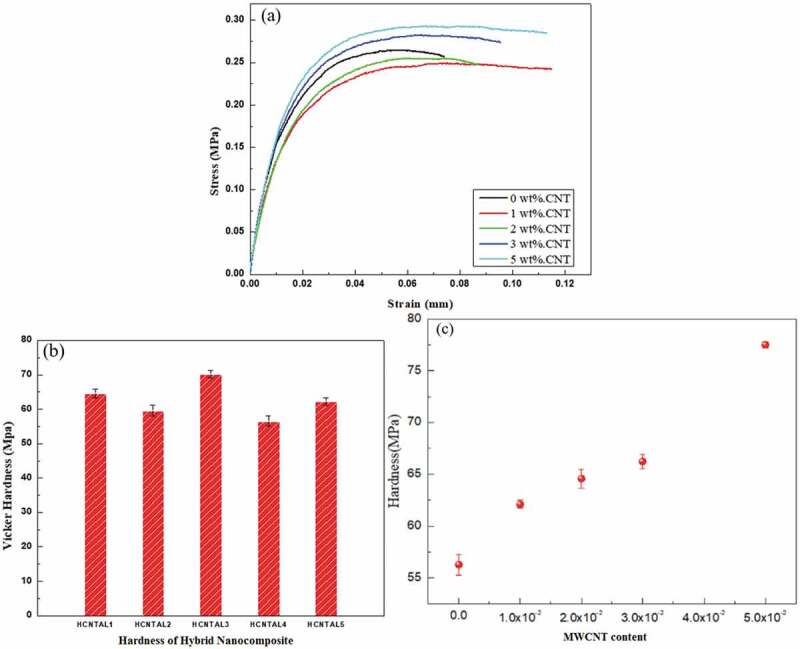
(a) Stress-strain curve for HDPE/MWCNT nanocomposite, (b) Hardness of hybrid Nanocomposite and (c) Linear equation of micro vickers hardness.

Figure 4(a) presents the stress-strain curve for HDPE/MWCNT/Al_2_O_3_ nanocomposite. It may be seen that the composite specimen exhibits the highest ultimate tensile stress at 0.3 MPa. The stress, as well as the strain of the nanocomposites with higher concentration of MWCNT, is more than the ones with lower concentrations. Higher stress may be attributed to the reinforcement of the polymer matrix and the exhibition of higher strain is complemented with the increase in ductility in the nanocomposites (Table 1) with high MWCNT content. This strain behavior is ascribed to the longitudinal orientation and the thread-like formation of the MWCNT, observed in the TEM pictures in Figure 3(b), which facilitates the effective transfer of stress from the matrix to the MWCNT [13,15,30]. High ductility coupled with higher stress (Table 1) has resulted in a higher strain. As examined from Figure 4(b,c), the Vicker’s hardness increases up to 37% with increasing the addition of MWCNTs compared to pure HDPE. The bulk hardness increases consistently with the addition of the MWCNTs. On closer examination of the stress-strain curve Figure 4(a), the elastic modulus of HDPE is more than 1% and 2% MWCNTs however, as the percentage of MWCNT is increased beyond 2%, the elastic modulus improves and exhibits higher value than HDPE. This might be credited to the narrowing of the interspaces between molecules and reducing their mobility. One of the best ways to reinforce mechanisms in polymer matrices is to transfer the shear stresses in the nanoparticle-matrix interface and to nucleate a 3D nanostructured network. It reduces the mobility of the polymer bonds, thus leading to changes in the elastic modulus of the nanocomposite [70,71].

**Table 1.** Mechanical properties of HDPE/MWCNT nanocomposite.

	Elastic modulus E (GPa)	Ultimate Tensile strength (MPa)	Max force (N)	Ductility % elongation	Toughness M J/m^−3^	Hardness (MPa)
(HDPE-0 wt.%)	0.74 ± 0.01	26.1	121.7 ± 2.5	7.40	160	56.2 ± 1.1
(HDPE-1 wt.%)	0.72 ± 0.03	24.2	115.5 ± 2.5	8.70	610	62.08 ± 1.8
(HDPE-2 wt.%)	0.75 ± 0.02	25.5	118 ± 0	9.54	400	64.5 ± 1.5
(HDPE-3 wt.%)	0.96 ± 0.04	27.2	126.3 ± 7.6	11.29	210	66.2 ± 1.07
(HDPE-5 wt.%)	1.02 ± 0.02	29.0	135.7 ± 4.5	11.48	250	78.2 ± 2

Table1 illustrates the mechanical properties of HDPE and nanocomposites i.e. elastic modulus, ultimate tensile stress (UTS), elongation, toughness, and hardness. The maximum value of elastic modulus reaches up to 1.02 GPa with 5% MWCNT, higher by 37%, compared with the pure HDPE. The 3% MWCNT nanocomposite exhibit higher elastic modulus than the pure HDPE while it is of the same order for 1% and 2% nanocomposite. The UTS first decreases up to 2% of MWCNT and then increases. The ductility also displays a consistent increase with the addition of MWCNTs. The toughness has been calculated from the area under the load stroke curve, the toughness of the pure polymer was about 160 MJm^−3^ which is less when 1% MWCNT was impeded into the polymer. However, the toughness decreased sharply with the increase of CNTs in the HDPE matrix probably due to the agglomeration of MWCNT in the HDPE matrix with increasing condensation of filler content. The hardness exhibits a consistent increase with the addition of the MWCNT.

Apart from the toughness, the other mechanical properties have marked improvement ascribed to reasonably good dispersion of MWCNT in HDPE polymer, evident from the SEM and TEM figures. According to recent research work, MWCNTs reinforcement mostly provides strength refinement, furthermore, it can also transfer their strength to composite materials by taking over the load applied to the matrix material. It has been confirmed that MWCNTs prohibit the propagation of cracks in the matrix during erosion situations through their foaming motion. This case is also confirmed by the literature. However, the strengthening can vary according to the reinforcement material but the MWCNTs generally give positive results toward the improvement of mechanical properties [72,73]. The mechanical properties of HDPE/MWCNT nanocomposites are enhanced due to effective CNTs reinforcement in the composites. The good dispensability of the MWCNT in the HDPE is a key factor for increasing the yield strength, ductility, and impact strength. The transfer of stress from the matrix to the reinforcement depends on the interfacial interaction, which was sound owing to the uniform dispersion. Nevertheless, the optimal MWCNT content in the HDPE matrix is important for maximizing the mechanical properties. As the amount of MWCNT increases beyond a certain value, the nanotubes tend to accumulate into clusters compromising the mechanical properties. The thread-like architecture of the MWCNT CNTs may develop a network-like structure by interweaving among themselves and forming Vander Waal and π bonds [74].

The mechanical properties of the composite strengthened with MWCNT along with nano Al_2_O_3_ According to the results also it has been found that the addition of alumina linearly increases the hardness particles are given in Table 2. The elastic modulus first increases and then reduces indicating that the nano alumina has a positive impact on elastic modulus but as the MWCNT increases, the combined effect of these reinforcements together doesn’t affect the elastic modulus. The maximum elastic modulus is displayed by 0.6 wt%CNT-2.4 wt%Al_2_O_3_/HDPE nanocomposite (1 ± 0.073 GPa). The same observation is recorded for UTS, it first improves slightly and then becomes stagnant. The maximum hardness is exhibited by 1.2 wt%CNT-1.8 wt%Al_2_O_3_/HDPE nanocomposite. It records a hardness of 70.20 ± 1.3 MPa marking an increase of 6% compared to 3 wt%MWCNT. Toughness and UTS of the composites containing 0.6 wt%CNT-2.4 wt%Al_2_O_3_ increased by 2.35 times and 2.9% compared to 3 wt%MWCNT. It is interesting to note that the improvement in ductility in the hybrid nanocomposites is much better than that in MWCNT nanocomposites. The presence of nano alumina particles may have reduced the agglomeration of the MWCNT in the polymer matrix, increasing ductility. For composites containing 1.8 wt%CNT-1.2 wt%Al_2_O_3_, the ductility increased by 145% and 141% compared with 3 wt% and 5 wt%MWCNT nanocomposites respectively.

**Table 2.** Mechanical properties of hybrid Al_2_O_3_/HDPE/MWCNT nano composite.

	Elastic modulus E (GPa)	Ultimate Tensile strength (MPa)	Max force (N)	Ductile % elongation	Toughness MJ/m^−3^	Hardness (HV_0.03_)
(0 wt%MWCNT, 3 wt%Al_2_O_3_)	0.97 ± 0.013	24.6	125.7 ± 1.53	23.2	569	64.58 ± 1.2
(0.6 wt%MWCNT, 2.4 wt%Al_2_O_3_)	1.00 ± 0.073	28.01	130.7 ± 9.61	25.5	705	64.58 ± 1.2
(1.2 wt%MWCNT, 1.8 wt%Al_2_O_3_)	0.97 ± 0.008	27.3	127 ± 1.00	19.79	399	70.20 ± 1.3
(1.8 wt%MWCNT, 1.2 wt%Al_2_O_3_)	0.95 ± 0.053	26.5	123.3 ± 6.81	27.7	604	56.62 ± 1.5
(2.4 wt%MWCNT, 0.6 wt%Al_2_O_3_)	0.97 ± 0.002	27.1	126.8 ± 0.29	17.9	369	62.25 ± 1.6

It has been found that adding aluminum oxide besides MWCNTs marked impressive improvements in mechanical properties, compared with 3/5 wt% MWCNTs/HDPE nanocomposites. The possible reason behind this observation is the homogenous dispersion of nanotubes into the polymer matrix in the presence of nano alumina particles. The addition of very small particles (nano Alumina) leads to the segregation of the particles in the polymer matrix. To retard the agglomeration of the particles, the capping agent (TWEEN) was used [75]. The addition of MWCNT and nano alumina produced fine nanocomposites. Usually, the interface between the matrix and the filler is the weakest link in the composite influencing the mechanical properties. The capping agent has helped to secure a strong linkage between the nano alumina and the HDPE matrix [76]. The hybrid nanofillers have resulted in better mechanical properties and supplemented better dispersion of each in the matrix.

### 3.3. TGA analysis of HDPE/MWCNT composites

**Figure 5.**
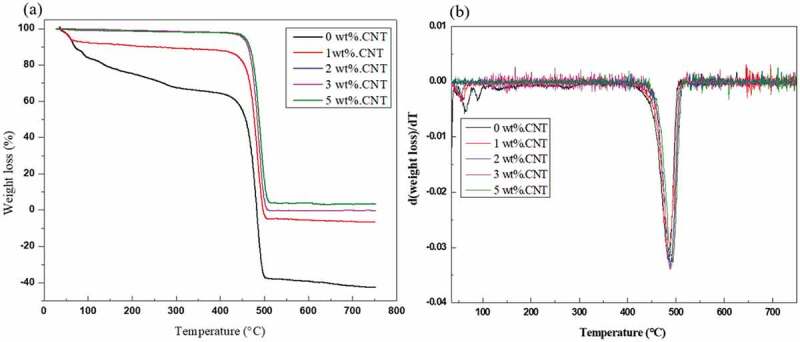
(a) TGA curves of MWCNT/HDPE nanocomposites and (b) DrTGA curve of MWCNT/HDPE nanocomposite.

TGA curves exhibit onset temperature dwindling with the addition of chemically treated MWCNT due to the presence of amorphous carbon in the MWCNTs. Mass loss degradation temperature of the nanocomposite increases as the MWCNTs ratio increases according to the TGA data as shown in Figure 5(a). This observation may be ascribed to the strengthening of the HDPE matrix by MWCNT, which has higher thermal conductivity and a favorable effect on the stability of nanocomposites [77].

The curves of all the materials exhibit similar shape ascribed to the same matrix material (HDPE). The lowest weight loss percentage is obtained for 3% and 5% MWCNTs/HDPE nanocomposites signifying that the mass loss melting speed rate decreases with increasing MWCNTs weight ratio and the melting point temperature increases as shown in Figure 5(b). The zigzag variation of the weight loss/temperature curve may be credited to the anisotropy of the material. As the content of MWCNT increased in the HDPE matrix, the anisotropy also increased, consequentially increasing the variation of the curve. The presence of MWCNT in the HDPE matrix has led to the non-homogenous distribution of the nanotubes in longitudinal and transverse directions, hence this anisotropy. Irrespective of the content of MWCNT, the weight loss curves of nanocomposites exhibit a similar trend, appearing above the HDPE due to the increased thermal conductivity.

### 3.4. Water absorption

**Figure 6.**
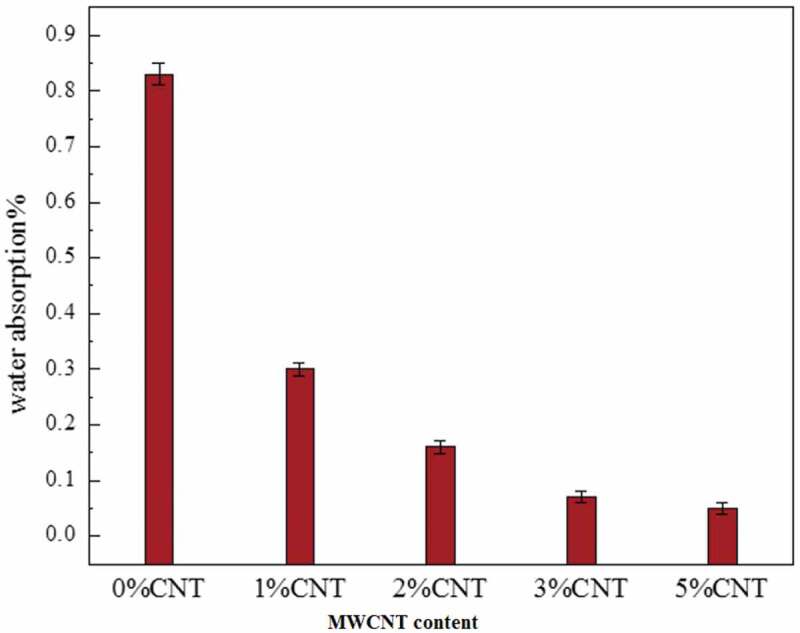
Results of water absorption.

One of the most important parameters for polymer nanocomposite is water absorption because it affects their life cycle [78,79]. The idea that cellulosic fibers easily absorb water is a major reason for fiber surface treatments. The particles treated by graphite fibers and its derivatives may absorb less amount of moisture, and thus support adhesion to the polymer matrix, which results in better performance in a humid environment. The high-water absorption of the polymer nanocomposite may cause difficulties during processing as well as in service. This can be due to partial curing of the thermosetting matrices, the presence of gaps or cracks, or even poor matrix–fiber adhesion [80]. It was observed that MWCNT/HDPE nanocomposites display less water absorption compared to that of pure HDPE, as shown in Figure 6. The inclusion of MWCNTs facilitated the crystallization of HDPE and therefore led to higher crystallinity in HDPE nanocomposites making it difficult for water to diffuse into the HDPE matrix.

### 3.5. Cytotoxic activity of Al_2_O_3_/HDPE/MWCNT composites

**Figure 7.**
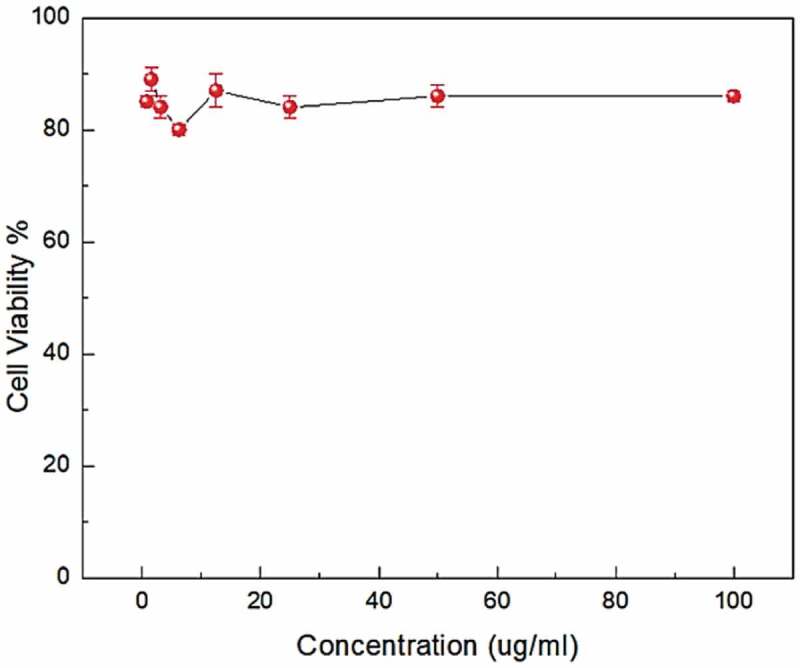
Cell viability of HDPE/1.2 wt%MWCNT/1.8 wt%Al_2_O_3_ nanocomposites over human cell lines.

The in-vitro cytotoxicity tests of HDPE/1.2 wt%MWCNT/1.8 wt%Al_2_O_3_ nanocomposites over human normal epithelial cell line 1- BJ1 (normal Skin fibroblast) are given in Figure 7. The test solution was prepared by using the hybrid composite with concentrations between 100 to 0.78 µg/ml and 100–75PPM MTT in separate iterations. This method is reliable and has good reproducibility [81]. The normal cells are very sensitive to any foreign material. The MTT assay probing system detected cell viability. During the initial (lower) concentration of the nanocomposites (<10 µg/ml) the cell viability varied between 79–90% as this concentration was not sufficient to provide exposure to the broader human cell sample. As the concentration of the nanocomposites increased beyond 10% and more, the cell viability stabilized around 83%. Higher the concentration of the nanocomposites, the better was the cell viability. According to the results, the majority of cells remains safe and viable under all concentration. The minimum cell viability was around 80%, which is considered as biocompatible.

For hip joint implants, the metallic and ceramic materials have been explicitly used but with limitations and a few hazards. A few polymers such as UHMWPE [2] and HA [8] have been tried with promising results. The HDPE in its pure form was not as successful as a potential material [24]. In the present study, the HDPE-MWCNT/Alumina nanocomposites have been developed and characterized for mechanical properties and cytocompatibility and the results are encouraging. These composites have emerged as a potential prosthetic implant material with less weight, less cost, and satisfactory biocompatibility. HDPE is a promising material; its nanocomposite has not been evaluated for prosthetic implants to date.

## 4. Conclusions

The metallic and ceramic materials have several disadvantages. Polymers in their pure form are not suitable for prosthetic implants. Nanotechnology and nanocomposites offer great potential in biomedical applications. The organic materials are more biocompatible. HDPE in its pure form has been successfully used as a prosthetic foot (external) but failed as an implant material due to limited mechanical properties. Alumina and MWCNT have been used as property enhancing fillers in certain biomedical nanocomposites. Hence an organic nanocomposite with HDPE and MWCNT/Nano-Alumina is selected as a potential material for a biomedical implant and its mechanical properties and biocompatibility have been discussed. HDPE/MWCNT-Alumina nanocomposites have not been explored for prosthetic implants. The HDPE polymer reinforced with MWCNTs and alumina by using a wet chemical approach to develop hybrid nanocomposites. Structure and morphology of the material have been characterized using SEM, TEM, and XRD. It has also been reported that there is a weak interface between 1, 2%wt MWCNT particles with the polymeric HDPE material, however good physical bonding of MWCNTs was observed when the ratio becomes 5%. Improvement in the mechanical properties of the material after incorporation of the MWCNT particularly with a ratio of 5%. The hybrid nanocomposites displayed better mechanical properties than the MWCNT/HDPE. TGA characterization of the prepared composite exhibits that mass loss melting speed rate decreases with increasing MWCNTs weight ratio and melting point temperature increases. The improvement in ductility in the hybrid nanocomposites is much better than that in HDPE/MWCNT nanocomposite. The water absorption studies displayed good performance with the lowest absorption (<0.1%) for 5% MWCNT compared to other ratios. As this work is going to be applied for medical applications, it is important to determine the biocompatibility of the hybrid nanocomposites. Cytotoxicity or cell cycle study of the normal cells has been done for this purpose and it shows low toxicity (<20%) against human normal cells. The results show that these composites are strong enough to bear the gait requirements of the hip joint and their cytocompatibility is satisfactory. According to these results, we can conclude that HDPE/MWCNTs/Al_2_O_3_ nanocomposite could be a good and promising candidate material for the total joint replacement and other structural biomedical applications. The authors suggest further studies on the tribological performance of the HDPE/MWCNTs/Al_2_O_3_ hybrid nanocomposites to characterize them comprehensively and approve them for biomedical implant material.

## Highlights

- Hip joint is a major load bearing joint and its deterioration is a common health issue.
- Metals, ceramics and polymers have limitations as prosthetic hip-joint material.
- HDPE/MWCNT/Al_2_O_3_ nanocomposites were explored for prosthetic hip-joint material.
- The nanocomposites exhibited better mechanical properties and biocompatibility.
- HDPE/0.6% MWCNT/2.4% Al_2_O_3_ emerged as the optimum composition.
